# Establishment and characterisation of testicular cancer patient-derived xenograft models for preclinical evaluation of novel therapeutic strategies

**DOI:** 10.1038/s41598-020-75518-3

**Published:** 2020-11-03

**Authors:** Gerda de Vries, Ximena Rosas-Plaza, Gert Jan Meersma, Vincent C. Leeuwenburgh, Klaas Kok, Albert J. H. Suurmeijer, Marcel A. T. M. van Vugt, Jourik A. Gietema, Steven de Jong

**Affiliations:** 1 Department of Medical Oncology, University of Groningen, University Medical Center Groningen, Hanzeplein 1, 9713 GZ Groningen, The Netherlands; 2 Department of Genetics, University of Groningen, University Medical Center Groningen, Groningen, The Netherlands; 3 Department of Pathology, Cancer Research Center Groningen, University of Groningen, University Medical Center Groningen, Groningen, The Netherlands

**Keywords:** Cancer models, Germ cell tumours, Testicular cancer

## Abstract

Testicular cancer (TC) is the most common solid tumour in young men. While cisplatin-based chemotherapy is highly effective in TC patients, chemoresistance still accounts for 10% of disease-related deaths. Pre-clinical models that faithfully reflect patient tumours are needed to assist in target discovery and drug development. Tumour pieces from eight TC patients were subcutaneously implanted in NOD scid gamma (NSG) mice. Three patient-derived xenograft (PDX) models of TC, including one chemoresistant model, were established containing yolk sac tumour and teratoma components. PDX models and corresponding patient tumours were characterised by H&E, Ki-67 and cyclophilin A immunohistochemistry, showing retention of histological subtypes over several passages. Whole-exome sequencing, copy number variation analysis and RNA-sequencing was performed on these *TP53* wild type PDX tumours to assess the effects of passaging, showing high concordance of molecular features between passages. Cisplatin sensitivity of PDX models corresponded with patients’ response to cisplatin-based chemotherapy. MDM2 and mTORC1/2 targeted drugs showed efficacy in the cisplatin sensitive PDX models. In conclusion, we describe three PDX models faithfully reflecting chemosensitivity of TC patients. These models can be used for mechanistic studies and pre-clinical validation of novel therapeutic strategies in testicular cancer.

## Introduction

Testicular cancer is one of the most common solid tumours in young men between 20–40 years of age and the incidence is rising worldwide^1^. TC can be divided in two subtypes, seminomas and non-seminomas, each accounting for approximately 50% of cases^2^. Seminomas resemble undifferentiated spermatogonia with low metastatic potential. Non-seminomas can be further divided in 4 histological subtypes displaying varying stages of differentiation. Embryonal carcinoma (EC) is the most undifferentiated type of non-seminomas, expressing various pluripotency markers^3,4^. Yolk sac carcinoma (YSC) and choriocarcinomas (CC) resemble extraembryonic differentiated tissues and express alpha-fetoprotein (AFP) and human chorionic gonadotropin, respectively^5^. The last non-seminoma subtype, teratoma, shows several patterns of somatic differentiation, which can be either incomplete (immature teratoma) or well-differentiated (mature teratoma)^6^. For non-seminomas, usually a mixture of histological subtypes is present, while tumours with pure histology are less frequent. Mixtures of both seminoma and non-seminoma components are also frequently observed.

TC is one of the few solid tumours that can effectively be treated with cisplatin-based chemotherapy. For metastatic disease, first-line chemotherapy results in cure rates of approximately 80%, whereas salvage therapy leads to curation in another 10% of patients. Based on serum marker levels, the IGCCC stratification classifies patients into poor risk patients who have a 5-year survival of only 50%^7^. Unfortunately, oncologists currently cannot reliably predict which patients will not respond to chemotherapy or will develop a relapse.

Preclinical testing of new therapeutic agents or combination strategies in testicular cancer is mainly performed in cell lines or cell line-based xenograft models. While cell lines have been used successfully for target discovery and mechanistic studies, they have limitations in the context of predicting responses to drug. The number of available TC cell lines is limited, with approximately 20 cell lines described in literature^8,9^. In addition, not all TC subtypes are well represented in these cell line models, with the majority of TC cell lines representing the EC subtype. To overcome these limitations, preclinical research is increasingly being performed using patient-derived xenograft (PDX) models. Advantages of PDX models have been described, including the histological preservation of the tumour when serially transplanted in different mice, and the molecular resemblance to the original tumour looking at genomic features and expression levels^10–15^. Various methods for establishing PDX models have been utilized, with differences in implantation site, tumour origin and mouse strain^16^. A limited number of TC PDX models has been established and described^17–20^. These TC PDX models, implanted either orthotopically or subcutaneously, were derived from either primary or metastatic tissue and represent all non-seminoma subtypes. Besides a histological assessment of tumour stability, the effects of serial passaging on the genetic and transcriptional level has not yet been determined.

Here, we studied the feasibility of establishing subcutaneous TC PDX models, and performed an extensive characterisation of these PDX tumours and their corresponding primary tumour tissues during serial passaging at the level of genes and proteins. Next, we tested sensitivity of these PDX models to cisplatin and two targeted drugs. Finally, we investigated the utility of TC-specific biomarkers in these models.

## Results

### Establishment and biobanking of testicular cancer PDX models

Between March 2016 and March 2019, tumour samples from eight patients were collected and implanted in male NOD-scid gamma mice, of which seven were diagnosed with TC (Table 1). Most tumours (7/8) were obtained via orchiectomy and implanted within four hours after surgery. One tumour (TC5) was stored overnight at room temperature in RPMI/10% FCS before implantation. PDX model TC4 was established from a needle biopsy of a metastatic tumour taken from a patient with refractory disease. From the tumour samples of these eight patients, in total three PDXs were successfully developed (38%) with an engraftment rate of individual tumour pieces of 69% (Table 1). The median latency between tumour implantation and tumour growth was 25 days (range 12–97), with large variation between tumours from different patients and between tumour pieces from individual patients (Table 1).

**Table 1:** Collection of GCTs used for PDX establishment received between March 2016 and March 2019.

Model	Sample	Site	Stage	Treatment^a^	Status at last follow up	Histology patient tumour	Tumour take rate (P0)	Tumour latency time (P0)
TC1	Surgery	IV (metastatic disease)	IV (metastatic disease)	Naive	Complete response	Mixed GCT: EC, YSC, TER, seminoma	3/6	12–97 days
TC3^b^	Surgery	Premalignant lesion	Premalignant lesion	N/A	N/A	GCNIS	N/A	N/A
TC4	Biopsy	II (metastatic disease)	II (metastatic disease)	CEB, TIP, TICE, carbo/pacli	Refractory disease	YSC	1/1	21 days
TC5	Surgery	IV (metastatic disease)	IV (metastatic disease)	Naive	Complete response	Mixed GCT: YSC, TER	5/6	15–29 days
TC6	Surgery	II (metastatic disease)	II (metastatic disease)	Naive	Complete response	Mixed GCT: Seminoma, EC, YSC, TER	0/6	N/A
TC7	Surgery	I (localized disease)	I (localized disease)	Naive	Complete response	Mixed GCT: EC, seminoma	0/6	N/A
TC8	Surgery	IV (metastatic disease)	IV (metastatic disease)	VIP	Complete response	TER	0/6	N/A
TC10	Surgery	II (metastatic disease)	II (metastatic disease)	Naive	Complete response	Seminoma	0/2	N/A

*GCT* germ cell tumour, *EC* embryonal carcinoma, *YSC* yolk sac carcinoma, *TER* teratoma, *GCNIS* germ cell neoplasia in situ.

*CEB* carboplatin, etoposide, and bleomycin containing chemotherapy; TIP paclitaxel, ifosfamide and cisplatin containing chemotherapy, *VIP* etoposide, ifosfamide and cisplatin and etoposide containing chemotherapy, *TICE* high-dose chemotherapy with stem cell transplant; carbo/pacli: carboplatin and paclitaxel containing chemotherapy.

^a^Treatment that patient had undergone before tumour tissue was collected for implantation.

^b^This tissue material was not included in take rate calculation since it was not a cancer lesion.

**Figure 1:**
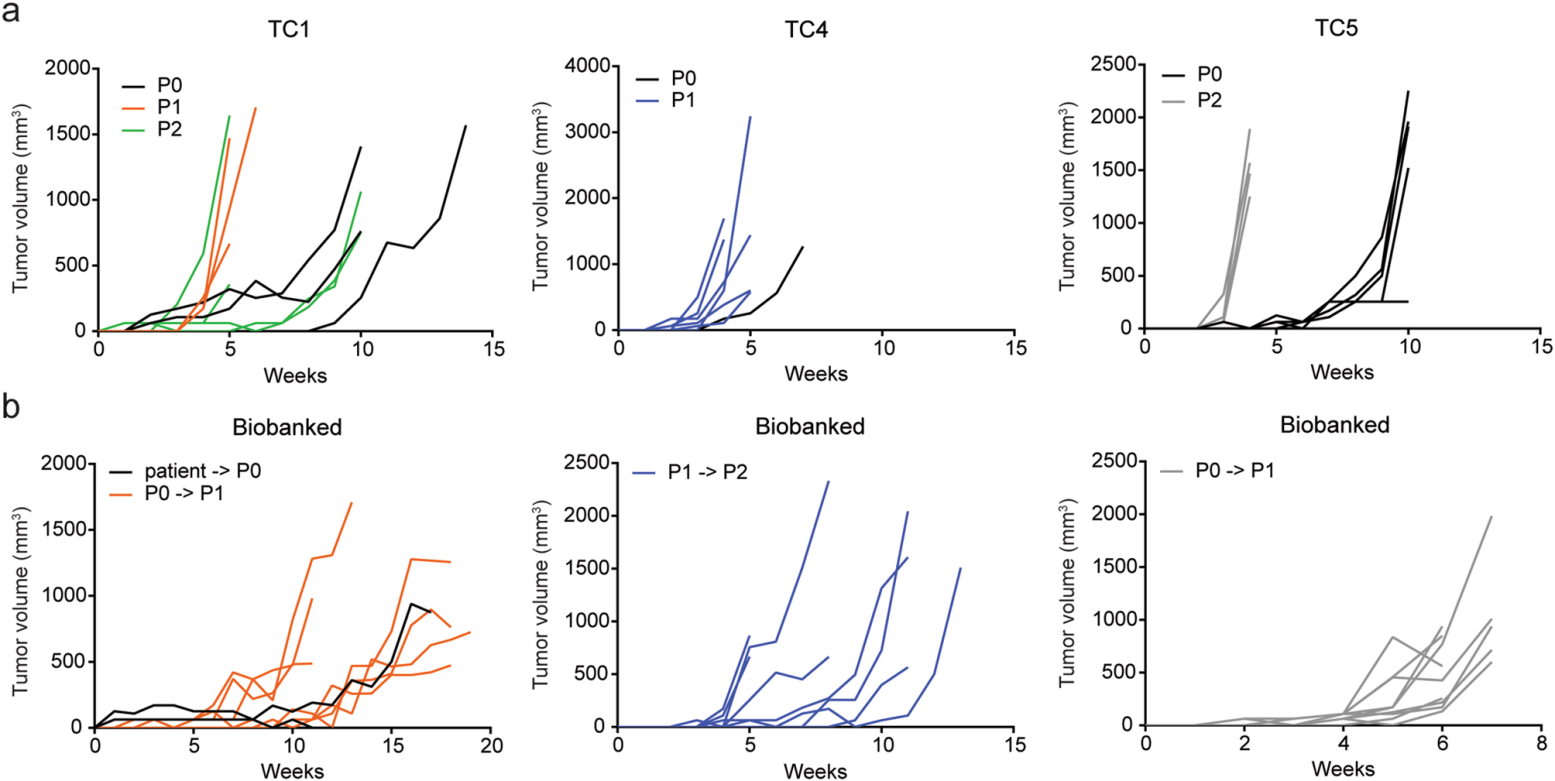
Growth curves of three TC PDX models and biobanking possibilities. (**a**) Tumour growth of freshly implanted tumour tissue of TC1 and TC4 in P0, P1 and P2 generations, and TC5 in P0 and P2 generations. (**b**) Tumour growth of primary and P0 material from TC1, P1 from TC4 and P0 material from TC5, stored in FCS/DMSO before (re-)implantation. Each line represents the individual tumour volume (mm^3^) of several mice.

Once first generation PDX models (P0) were established, serial passaging to second (P1) and third generation (P2) was successful for all three models (Fig. 1a). Overall, tumour growth was observed within 15 weeks after implantation for the three PDX models and their different passages. The effect of biobanking on tumour growth and take rate was evaluated at different generations (Fig. 1b, Suppl. Table 1). The specimen from TC1 showed tumour growth after patient material was biobanked in FCS/DMSO. Thawed patient material was able to engraft into P0 (1/2 mice) and thawed P0 material in P1 (5/5 mice) generation. Tumour take rates were 25% with 1 out of 4 implanted tumour pieces showing growth for P0 and 70% (7 out of 10 tumour pieces) for P1 generation. Latency of stored material for this model (7 days for P0 and 25 days for P1), was comparable to freshly implanted material (12 days for P0 and 35 days for P1 generation) (Suppl. Table 1). For TC5, frozen P0 tumour tissue was re-implanted in a second generation of mice (P1) and tumour growth was observed in 5/5 mice, with 9/10 pieces showing growth with a take rate of 90% (Suppl. Table 1). Biobanked P1 tumour tissue from TC4 was re-implanted into a third generation of mice (P2) and tumour growth was observed in 7/7 mice, with 8/14 pieces showing growth with a tumour take rate of 57% (Suppl. Table 1).

### PDX models partly retain immunohistochemical characteristics of the primary tumour

**Figure 2:**
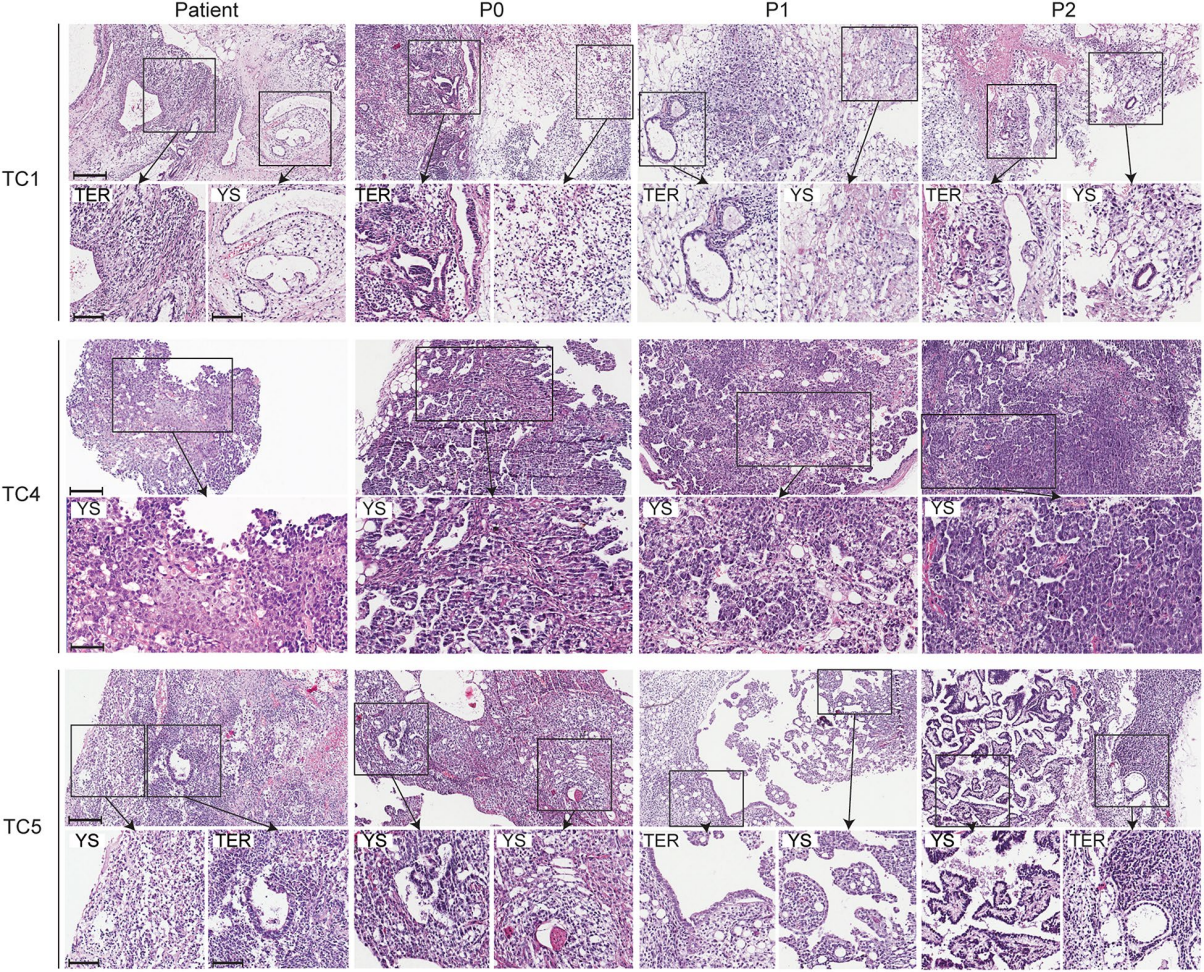
Histopathological characteristics of three established TC PDX models. HE staining at 10X and 20X magnification of patient tissue and tumours belonging to P0–P2 generations of PDX TC1, TC4 and TC5. 20 × magnification fields highlight the teratoma (TER) and yolk sac (YSC) components of TC1 and TC5 and the yolk sac histology of TC4. Mixed histology was conserved across all passages of TC1 and TC5 PDX models. Scale bars in the upper panels represent 200 µm, and scale bars in the smaller inserts represent 100 µm. Tumour histology: TC1 − mixed tumour (YSC + immature TER), TC4 − YSC, TC5 − mixed tumour (YSC + immature TER).

Histology of the primary patient tumour and three subsequent PDX generations was determined by H&E staining (Fig. 2). PDX models TC1 and TC5 originated from mixed germ cell tumours (Table 1). Histology of the patient tumour TC1 reported by pathology mentioned YSC, EC, teratoma and seminoma components. However, histological examination of the patient tumour pieces used for implantation showed YSC and immature teratoma components only, suggesting a sampling bias from the patient tumour. After implanting the primary tumour into mice, the histological subtypes YSC and immature teratoma remained in P0, as well as in the P1 and P2 generation. TC4 originated from a pure YSC and histology was retained over three subsequent passages. Patient tumour of TC5 consisted of YSC and immature teratoma components, subtypes that were retained in subsequent passages P0, P1 and P2. Based on observations with these three PDX models, we conclude that passaging of TC tumours in mice does not have major effects on histological subtype representation.

**Figure 3:**
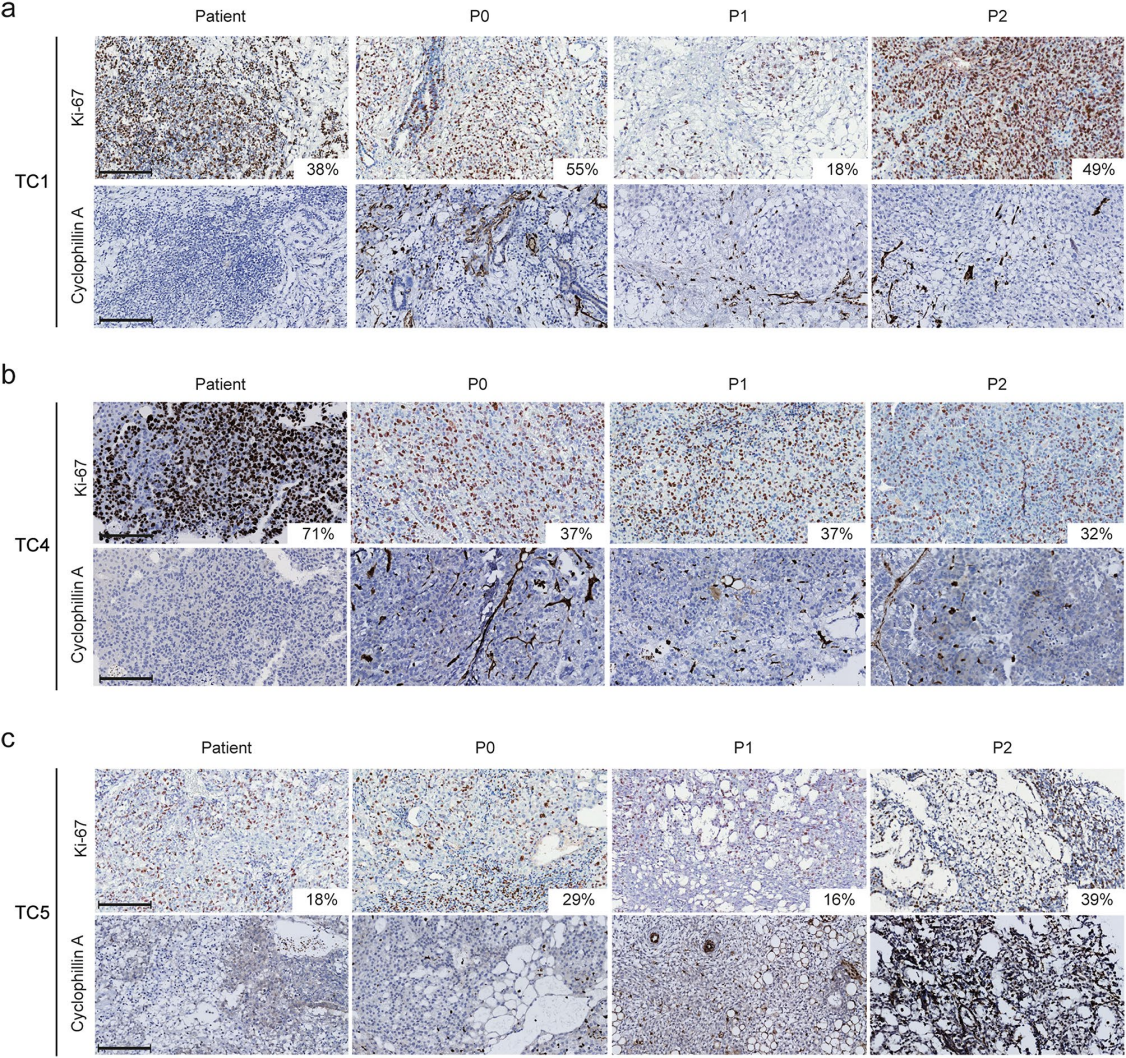
Proliferation index and mouse specific tumour infiltration across several passages. Images at 20X magnification of Ki-67 and cyclophilin A (mouse specific) IHC staining performed with patient and PDX tumours of TC1 (**a**), TC4 (**b**) and TC5 (**c**). Insertions in Ki-67 staining state proliferation index of each tumour. Scale bars represent 100 µm. Tumour histology: TC1 − mixed tumour (YSC + immature TER), TC4 − YSC, TC5 − mixed tumour (YSC + immature TER).

Tumours were stained for Ki-67, a marker of proliferation. All three PDX models showed high percentage of Ki-67-positive cells, which remained relatively constant over the three passages (Fig. 3a–c). Infiltration of murine stroma cells into the PDX tumours was assessed by staining for cyclophilin A, a protein involved in protein folding and recently identified as a sensitive target for detection of murine microenvironment in human tumour xenografts, using a mouse selective antibody^21,22^. The primary patient tumours from TC1, TC4 and TC5 were indeed negative for cyclophilin A, whereas mouse-specific infiltration was observed in all three PDX generations (P0–P2) of TC1, TC4 and TC5 (Fig. 3a–c).

Five patient tumour samples failed to engraft, one of which was classified as a germ cell neoplasia in situ, a pre-malignant lesion. Other patient tumour samples consisted of either mixed tumour histology, pure teratoma or seminoma components (Table 1, Suppl. Fig. 1). Proliferation indices of these tumours were determined and showed that all samples contained Ki-67 positive cells ranging from 11–61% (Suppl. Fig. 1).

### Genomic analysis of TC PDX models

**Figure 4:**
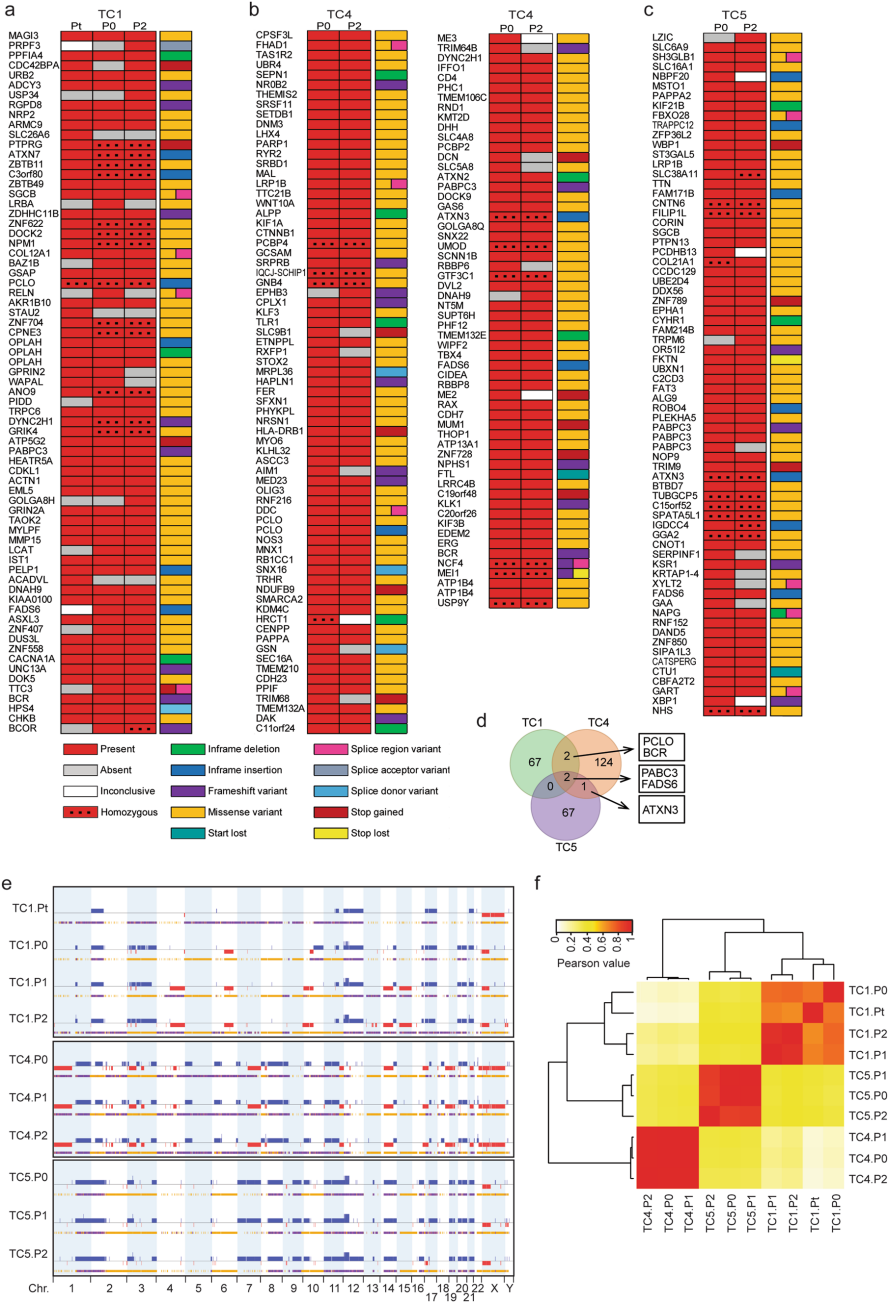
Somatic mutations and copy number alterations across different PDX passages. (**a**–**c**) Somatic mutations identified in the primary tumour (Pt), first generation (P0) and third generation (P2) PDX tumours of TC1, TC4 and TC5 detected by whole exome sequencing. Classification of mutations is indicated by the colour scheme in the legend. (**d**) Venn diagram showing number of shared mutations between PDX models, and corresponding genes. (**e**) CNA plots of PDX TC1, TC4 and TC5 representing the genome wide location of gains (blue) and losses (red) in primary tumour (Pt), and/or 3 subsequent PDX generations (P0, P1 and P2). The coloured bar below each CNA plot indicates loss of heterozygosity (yellow) or allelic imbalance (purple). (**f**) CNA concordance analysis between patients and different passages of PDX tumours by hierarchical clustering. Tumour histology: TC1 − mixed tumour (YSC + immature TER), TC4 − YSC, TC5 − mixed tumour (YSC + immature TER).

Whole-exome sequencing was performed on patient tumours (if available) and corresponding P0 and P2 passage PDX tumours. Calculation of the relative amount of human and mouse DNA, based on the percentage unambiguous human reads, showed that the majority of reads (91.7–99.7%) were human (Suppl. Table 2). Sequencing data revealed that all three TC PDX models contained wild type *TP53*. A total number of 71 non-synonymous somatic mutations identified in PDX TC1, 129 in PDX TC4, and 70 in PDX TC5 met our filtering criteria (Fig. 4a–c). Of these mutations, 75% was shared between patient and corresponding PDX tumours for TC1. Four unique mutations were observed in the patient tumour of TC1, which were not observed in the PDX tumours, and six mutations were observed in the PDX tumours only (Fig. 4a). For TC4 and TC5 respectively, 89% and 86% were shared between P0 and P2 generation PDX (Fig. 4b,c). These data showed that many somatic mutations observed in the P0 generation PDXs were retained in their corresponding P2 generation PDX, and that during the grafting process not many new mutations were accumulated, demonstrating genetic stability after passaging. Several interesting mutations were identified in our PDX models. For example, a missense mutation in *DOCK2* was observed in TC1, as well as a frameshift mutation in *BCR* that was also present in TC4. Other mutations observed in TC4 included *WNT10A* and *CTNNB1,* proteins involved in Wnt/β-catenin signalling pathway. In addition, two retinoblastoma-binding proteins were found to be mutated: *RBBP6* and *RBBP8/CtIP*. For TC5, a missense mutation was found in *CBFA2T2*. A complete list of all mutations and their predicted consequence on protein level are given in Supplementary Table 3. Several genes (including *BCR*, *PCLO*, *ATXN3*, *PABPC3*, and *FADS6*) were mutated in two or more PDX tumours (Fig. 4d). Furthermore, mutations were observed in *OPLAH*, *PABPC3*, *FADS6* and *CATSPERG* genes that are specifically expressed in testis tissue (The Human Protein Atlas). None of the mutations identified in our PDX models corresponded to recurrent mutations previously observed in TC^23–25^. We searched for GO terms associated with the identified somatic mutations per tumour model but no significant results were obtained. Based on the exome sequencing data, we investigated the copy number status of several genes previously associated with TC, or involved in PI3K/AKT/mTOR and p53 signaling. We observed copy number gains of *KRAS*, *MDM2* and *MYCN* in TC1 and TC5, but not in TC4. Copy number gain of *PIK3CA* was present in all three PDX models, as well as *AKT1* in TC1 and TC4 PDX tumours. Interestingly, copy number loss of *TP53* was observed in TC4 and TC5 PDX tumours. The genes *KIT*, *MTOR*, *TSC1* and *TSC2* were diploid in all samples.

Genome-wide copy number alterations (CNAs) were measured at different passages of PDX models, and on primary patient tumour pieces if available. Most TCs are aneuploid and are characterized by large scale copy number gains and losses^26–28^. A well-known anomaly is the 12p isochromosome, present in more than 80% of TC tumors^29^. Consistently, 12p copy number gain was present in TC PDX models TC1 and TC5. All models contain genomic segments that are overrepresented or underrepresented (Suppl. Table 4). These include losses on chromosome 4 (TC4) and gains on chromosomes 7 (TC5), 21 (TC1) and X (TC1, TC4), consistent with TCGA profiles and previous studies^25,30^.

The patient tumour of PDX TC1, containing around 50% healthy tissue, did not show many gains and losses, whereas considerably more gains and losses were observed in subsequent passages P0, P1 and P2, suggesting tumour cell enrichment and clonal selection bias. CNAs observed in the patient’s primary tumour were retained in the different generations of PDX TC1. Additional CNAs observed in the P0 generation, but not in the primary tumour, were retained in the subsequent P1 and P2 generation. Unfortunately, no DNA was available from the primary tumours of TC4 and TC5 to be able to assess changes in CNAs between patient and P0 generation tumours. For both TC4 and TC5, the P0 PDX tumours showed a genome-wide distribution of CNAs that was largely similar to those in the P1 and P2 generation (Fig. 4e). Overall, there was a high level of consistency regarding the total distribution of CNAs through serial in vivo passaging, indicating genomic stability of TC PDX tumours.

The correlation between different PDX passages was determined based on categorized CNA values of all SNP probes (n = 691,687). Heterogeneity was observed between the three different models. A strong correlation was observed between generations within each PDX model with Pearson r > 0.68 for TC1, r > 0.98 for TC4 and r > 0.84 for TC5. Correlation of the primary tumour of TC1 with its subsequent PDX passages was moderate with Pearson r between 0.57 and 0.64. Hierarchical clustering revealed that passages within a PDX model cluster more closely together than unrelated samples (Fig. 4f).

### Transcriptional profiles of TC PDX models

**Figure 5:**
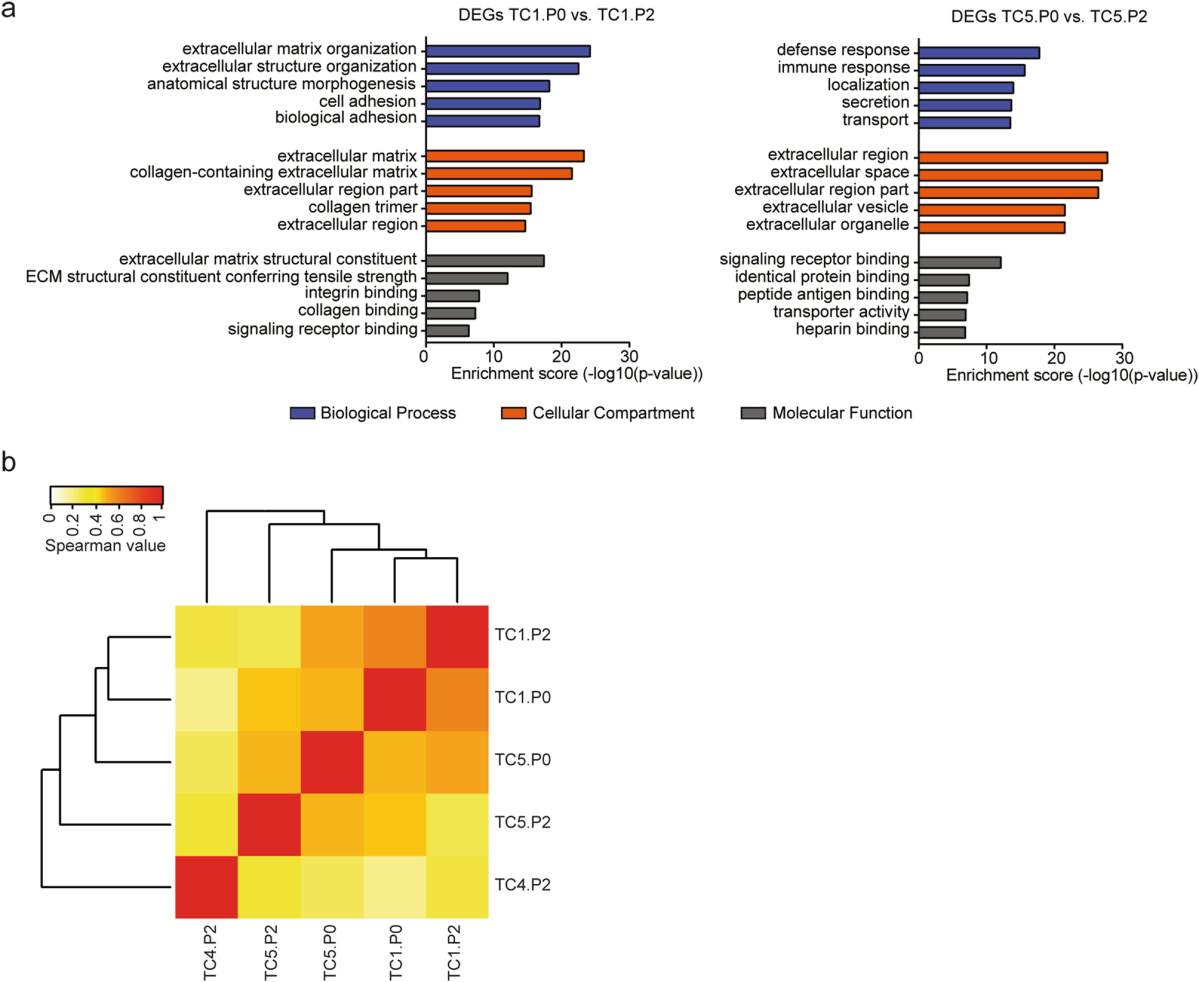
Differentially expressed genes in different PDX generations. (**a**) Gene Ontology (GO) analysis of significantly differentially expressed genes between the P0 and P2 generation PDX tumours of TC1 or TC5. (**b**) Relative distances by hierarchical clustering based on significantly DEGs between all samples. Tumour histology: TC1 − mixed tumour (YSC + immature TER), TC4 − YSC, TC5 − mixed tumour (YSC + immature TER).

Transcriptome analysis (RNA-seq) was performed on P0 and P2 generation PDX tumours of TC1 and TC5. Of TC4, only the P2 generation was included after RNA quality control. In total, five tumours were analysed by RNA-sequencing. Differential expression analysis was performed on paired PDX tumours of generation P0 and P2. A high concordance between paired PDX tumours was observed, while 211 genes were significantly differentially expressed for TC1 and 201 genes were significantly differentially expressed for TC5. Gene ontology analysis on DEGs between paired tumours (P0–P2) from TC1 and TC5 showed enrichment of genes that are mainly involved in the extracellular environment, including extracellular matrix organization, cell adhesion, secretion and immune response (Fig. 5a). These findings suggest that the human immune and stromal components are lost with increasing passaging in vivo, consistent with cyclophilin A staining of these tumours.

To gain insight into the differences between and also within tumours, correlation analysis on all DEGs was performed. Within TC1 and TC5, a moderate correlation between P0 and P2 was observed with Spearman r > 0.48. Weak to moderate correlation was observed between the P2 tumours of TC1, TC4 and TC5. Hierarchical clustering on DEGs of all samples revealed that P0 and P2 tumours from TC1 clustered together, but not P0 and P2 tumours from TC5. The largest correlation distance between all samples was observed for PDX TC4, which was established from a cisplatin-refractory patient (Fig. 5b). These data emphasize that the transcriptional profiles of different PDX passages are highly concordant.

### PDX models mimic response to conventional chemotherapy in patients

**Figure 6:**
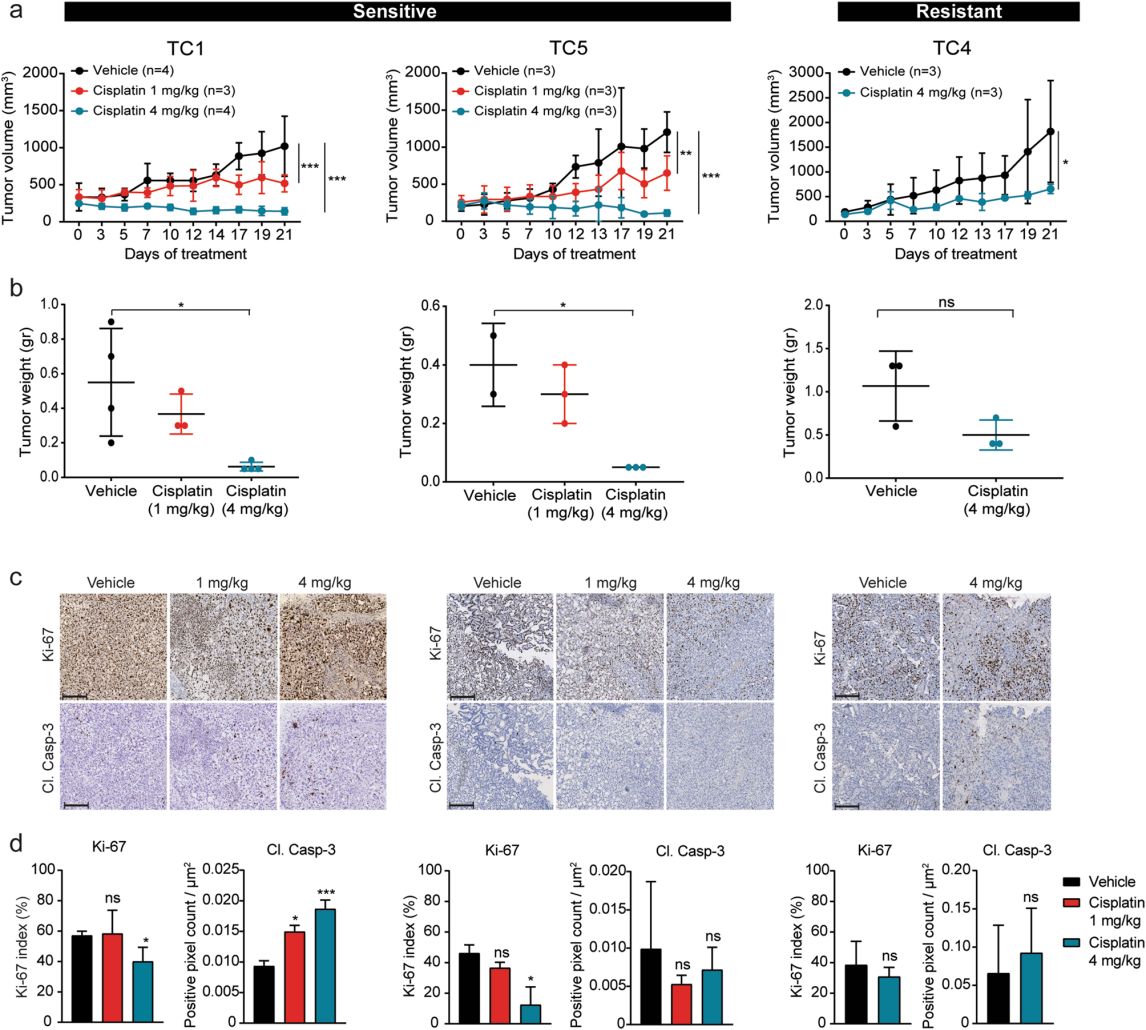
Cisplatin sensitivity of three TC PDX models. (**a**, **b**) Tumour growth, and final tumour weight of PDX models TC1, TC4 and TC5 treated with vehicle or cisplatin. Data shows average ± SD. (**c**) Representative images from tumours shown in (**a, b**) at 10X magnification. (**d**) Quantification of Ki-67 and cleaved caspase-3. Scale bars represent 200 µm. Tumour histology: TC1 − mixed tumour (YSC + immature TER), TC4 − YSC, TC5 − mixed tumour (YSC + immature TER).

Cisplatin is the main chemotherapeutic agent in the treatment of testicular cancer. We assessed whether cisplatin sensitivity of the established PDX models corresponded to the patients’ response. TC1 and TC5 were obtained from patients who had a complete response after cisplatin-based chemotherapy, while TC4 was obtained from a patient refractory to cisplatin treatment. A relatively low dose of cisplatin (1 mg/kg) resulted in a statistically significant tumour growth delay of both TC1 and TC5 (Fig. 6a), while minor differences were observed in tumour weight. Treatment with a high dose of cisplatin (4 mg/kg) completely abolished tumour growth of TC1 and TC5 (Fig. 6a). Tumour weight at the end of treatment was significantly lower in the high dose cisplatin group compared to vehicle treatment (Fig. 6b). Treatment of TC4 with a high dose of cisplatin (4 mg/kg) resulted in slightly delayed tumour growth over the course of treatment compared to vehicle controls (Fig. 6a). In line with this finding, no statistically significant difference was observed in TC4 tumour weight (Fig. 6b). The cisplatin-sensitive PDX tumours (models TC1 and TC5) showed a decrease in Ki-67 positive cells with increasing cisplatin concentrations, while no change in Ki-67 positivity was observed for the cisplatin-resistant model TC4. An increase in cleaved caspase-3 staining, an established marker of apoptosis, after cisplatin treatment was only observed in TC1 (Fig. 6c,d). Body weight of the male NSG mice was monitored during the course of treatment to measure treatment related toxicity. Mice in the group receiving the highest dose of cisplatin (4 mg/kg) showed significant weight loss at the end of treatment (Suppl. Fig. 2).

### AFP levels do not correlate with response to chemotherapy

The serum protein biomarker AFP is clinically used to assist diagnosis of TC and to monitor patients during follow-up. AFP is mainly expressed by the histological subtype YSC, although it can also be produced by EC component. To determine whether AFP was excreted by TC PDX tumours and might be used as a marker of tumour load, blood samples were collected at start and end of treatment. AFP was not detected in sera from mice without PDX tumours (data not shown). AFP was detected in all PDX-bearing mice at start of treatment (Suppl. Fig. 3a,b). Remarkably, AFP levels were lower or undetectable in the vehicle groups of TC1 and TC4 compared to the AFP levels at start of treatment, even though tumour volume increased. In contrast, serum AFP levels were higher after high dose cisplatin treatment in TC1 despite the strong decrease in tumour volume in all mice (Suppl. Fig. 3a). In the cisplatin-resistant TC4 model even though tumour volumes had increased at the end of treatment with a high dose of cisplatin, AFP levels were lower (Suppl. Fig. 3b). In addition, AFP expression was analysed on paraffin-embedded tumour material at the end of treatment. Vehicle-treated TC1 and TC4 tumours still expressed AFP and the intensity of AFP staining in both models had not changed after treatment with 4 mg/kg cisplatin (Suppl. Fig. 3c,d). Decreased abundance of AFP in the mouse serum of TC4-bearing mice after cisplatin treatment (4 mg/kg) can therefore not be explained by loss of AFP expression in the tumour.

### TC PDX models are sensitive to mTORC1/2 and MDM2 inhibition

**Figure 7:**
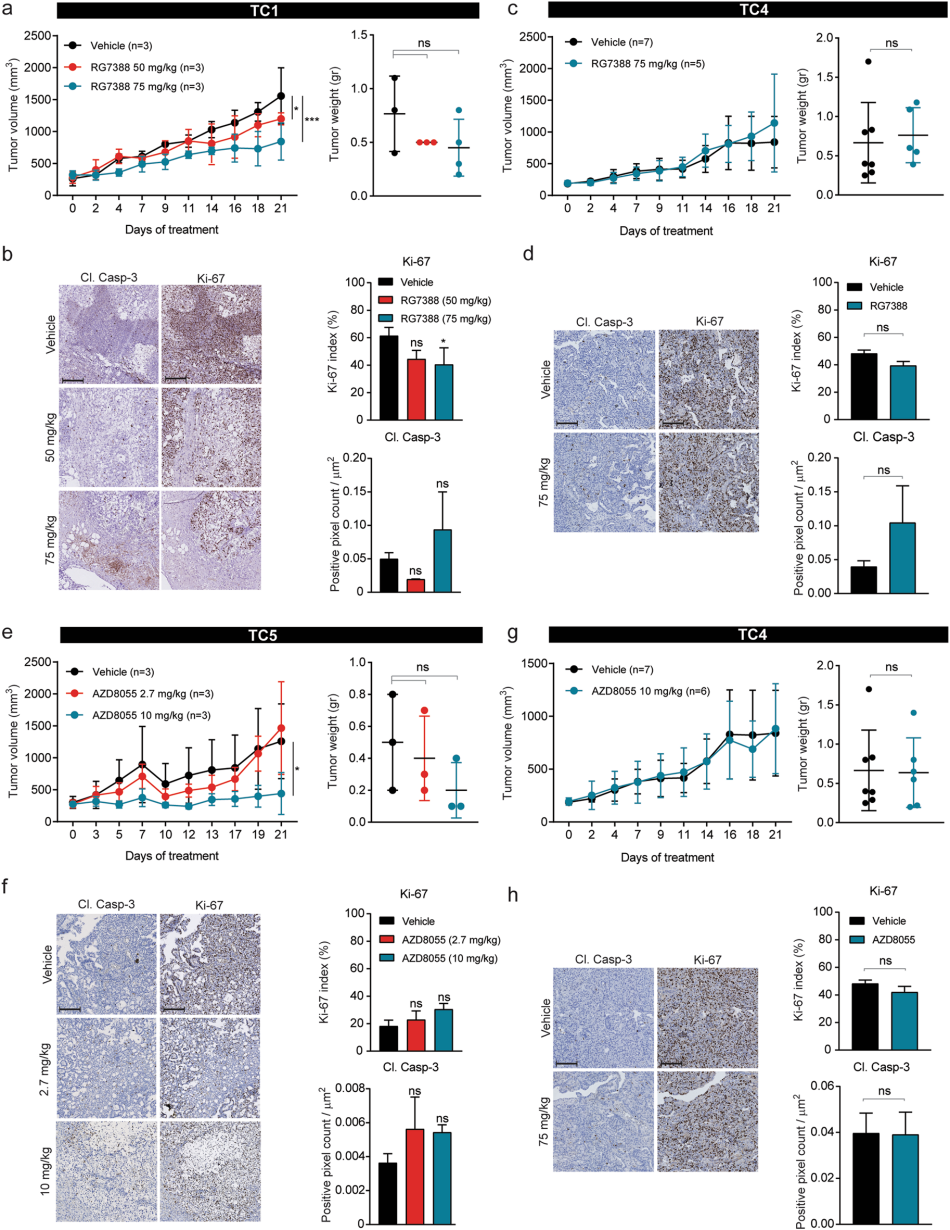
Sensitivity of TC PDX models to novel targeted drugs. (**a**) and (**c**), Tumour growth, and final tumour weight of PDX TC1 and TC4 treated with vehicle or RG7388. Data shows average ± SD. (**b**) and (**d**)**,** Representative images of tumours shown in (**a**) and (**c**) respectively at 10X magnification, scale bars represent 200 µm, and quantification of Ki-67 and cleaved caspase-3. Data shows average ± SEM. (**e**) and (**g**)**,** Tumour growth, and final tumour weight of PDX TC5 and TC4 treated with vehicle or AZD8055. Data shows average ± SD. (**f**) and (**h**)**,** Representative images of tumours shown in (**e**) and (**g**) respectively at 10X magnification, scale bars represent 200 µm, and quantification of Ki-67 and cleaved caspase-3. The same tumour volume and tumour weight of TC4 vehicle treated mice are shown in (**c**) and (**g**), as all treatment groups were included in a single experiment. Tumour histology: TC1 − mixed tumour (YSC + immature TER), TC4 − YSC, TC5 − mixed tumour (YSC + immature TER).

Preclinical efficacy of two novel targeted agents for TC was assessed in TC1, TC4 and TC5 PDX models. Targeting MDM2 has shown promising results in several TC cell lines. P53 activity is regulated by MDM2, an E3 ubiquitin ligase. Binding of MDM2 to the transactivation domain of p53 prevents the transcriptional activity of p53^31^. Blocking the interaction between MDM2 and p53 using nutlin-3a, a small molecule inhibitor of MDM2, is of potential therapeutic value as it leads to activation of p53 and induces a p53-dependent apoptotic response in TC cell lines^32,33^. As most TC tumours contain wild type *TP53*, including all our TC PDX models, the efficacy of RG7388, another MDM2 inhibitor with superior potency and selectivity compared to nutlin-3a, was tested in two different doses in TC1. Only little effect on tumour growth was observed when RG7388 was applied in low doses (50 mg/kg) (Fig. 7a). Treatment with a higher dose of RG7388 (75 mg/kg) resulted in considerably smaller tumour volumes compared to the vehicle-treated group. Small but non-significant differences in tumour weight were observed (Fig. 7a). IHC analysis of the tumours showed a trend towards lower Ki-67 positivity in both RG7388 treatment arms (Fig. 7b). Furthermore, an induction of cleaved caspase-3 score was observed in tumours treated with the high dose of RG7388 (75 mg/kg) (Fig. 7b). In TC4, only the highest dose of RG7388 (75 mg/kg) was tested. No effect was observed on tumour growth and tumour weight (Fig. 7c). Lack of sensitivity to MDM2 inhibition could indicate the presence of a *TP53* mutation, however whole-exome sequencing data revealed that all three TC PDX models contained wild type *TP53*. Ki-67 analysis showed no difference between control and RG7388 treated tumours. An increase in cleaved caspase-3 positivity was detected in tumours treated with RG7388 (Fig. 7d).

Another potentially effective therapeutic target for TC is mTOR, as the PI3K/AKT/mTOR pathway was shown to be highly active in TC models^19,34–37^. In addition, copy number gain of *PIK3CA* was present in all samples, as well of *AKT1* in TC1 and TC4 PDX tumours. Previously, we demonstrated that TC cell lines are highly sensitive to the mTORC1/2 inhibitors AZD8055 and MLN0128^37^. The mTORC1/2 inhibitor AZD8055 was tested in two PDX models and the effect on tumour growth was assessed. Two dosages of AZD8055 were assessed in TC5, where the lower dose (2.7 mg/kg) had no effect on tumour growth and tumour weight (Fig. 7e). In this PDX model, delayed tumour growth, reflected by lower tumour weight, was observed in mice treated with the highest dose of AZD8055 (10 mg/kg) compared to the vehicle group. In contrast, no effect of the highest dose was observed in the cisplatin-resistant PDX model TC4 (Fig. 7g). IHC staining of TC4 and TC5 showed that increasing concentrations of the mTORC1/2 inhibitor did not induce any changes in Ki-67 positivity or cleaved caspase-3 positivity (Fig. 7f,h). Mouse weights, monitored during the three week treatment period, were not affected by RG7388 or AZD8055 treatment (Suppl. Fig. 4).

## Discussion

In the present study, we describe the development of subcutaneous TC PDX models. Tumour material from TC PDXs could be efficiently biobanked, which facilitates their use for future experiments. In addition, in depth characterisation of three TC PDX models indicates that PDX tumours have retained important patient germ cell tumour characteristics, including (mixed) tumour histology and sensitivity to conventional chemotherapy. The observed sensitivity to the MDM2 inhibitor RG7388 and the mTORC1/2 inhibitor AZD8055 in TC PDXs highlights the potential of PDX models to test new targeted treatment strategies for TC.

It has been proposed that the testis niche is necessary for tumour establishment^16^. Although orthotopic tumour implantation may be favourable, the implantation is more laborious and complex compared to subcutaneous implantation. Moreover, monitoring of tumour growth requires imaging techniques and luciferase expressing tumours. Here, we have shown that the engraftment rate of subcutaneously implanted TC tumours from different patients is 38%. Others reported engraftment rates of 35% for orthotopically implanted TC PDX models, and 25% for subcutaneously implanted TC tumours^20,38^. Albeit based on a limited number of TC PDX models, these results suggest that the site of implantation is not a major determinant of successful engraftment.

Histological evaluation showed that one model was of pure-YSC histology and two models had a mixed tumour histology, with YSC as the major component. Interestingly, patients with non-seminoma tumours containing the YSC component have been reported to have poorer prognosis than those without the YSC component^39^. Our data indicate that the non-seminoma components are retained within subsequent PDX passages. These models show high genetic and transcriptional stability over time. Most mutations detected with whole-exome sequencing in the P0 generation PDXs are retained in their corresponding P2 generation. At the transcriptional level PDX tumours also remain stable with increasing passages, where DEGs show enrichment of genes mainly involved in the extracellular environment, which most likely indicates a loss of human stromal components, consistent with an increase in mouse cyclophilin A staining of these tumours. Copy number variations in first passage PDX tumours (P0) are retained in the P1 and P2 generation. Frequently, CNAs were observed in PDX tumours of TC1 that were hardly or undetected in the primary tumour. This can partly be explained by human tumour cell enrichment as the primary tumour contained around 50% healthy human tissue, which is now substituted by normal mouse tissue. However, a clonal selection bias cannot be ruled out. Previously, it has been described that the acquisition of CNAs during PDX passaging deviates from the acquisition of CNAs during tumour progression in patients, indicating mouse specific tumour evolution^40^. Therefore, to maintain genetic resemblance with patient tumours as close as possible, it is important to use low passage-PDX tumours when studying drug-sensitivity for example. Even though clonal selection has been found to occur after PDX establishment^40,41^, genomic landscape analysis still shows higher resemblance of PDX models to human tumours than cell line models^42^.

Among the identified somatic mutations, several are present in tumour suppressor genes and oncogenes. For example, a frameshift mutation in *BCR*, present in both TC1 and TC4 models, has previously been observed in a non-seminoma TC patient tumour (cBioportal). Furthermore, in TC1 a mutation is observed in *DOCK2*. Both DOCK2 and BCR are involved in activation of RAC1, a Rho family GTPase with significant homology to RAS, which plays a role in cell proliferation and drug resistance^43^. Two mutations present in TC4 include *RBBP6*, a protein which promotes degradation of p53^44^, and *RBBP8/CtIP*, a protein involved in double-strand break repair by homologous recombination^45^. The latter mutation is present in the C-terminal domain, important for binding to a DNA damage sensor protein complex involved in homologous recombination^46^. Interestingly, loss of RBBP8/CtIP has been associated with deficient DNA double strand break repair, and subsequently with PARP inhibitor sensitivity^47^. In TC5 a mutation is present in *CBFA2T2,* a transcriptional co-repressor regulating and interacting with the germline-specific transcription factor PRDM14 regulating pluripotency and germline development. CBFA2T2 has been shown to stabilize PRDM14 and OCT4, a biomarker of the EC subtype, on chromatin via its oligomerization, allowing for stable transcription factor binding^48^.

Here, we show that all three TC PDX models possess comparable cisplatin sensitivity as their corresponding patients, adding to growing evidence that PDX models are superior in predicting drug response in the clinic as compared to cell line models^42,49,50^. We evaluated the possibility to study known biomarkers of TC to monitor treatment response in PDX models. The inverse relation between AFP serum levels and tumour volume or response to cisplatin treatment in both PDX models suggests that AFP should not be used as response evaluation tool in TC PDX models.

Inhibition of the MDM2–p53 interaction, using the MDM2 inhibitor nutlin-3a, has been shown to result in hyperactivation of the p53 pathway combined with a strong induction of apoptosis in TC cells^33^. Additionally, we have identified AKT and S6 to be among the top phosphorylated proteins in TC cells, which are part of the PI3K/AKT/mTORC pathway^37^. To that end, we have tested two clinically relevant targeted drugs, the MDM2 inhibitor RG7388 and the mTORC1/2 inhibitor AZD8055 using wild type p53 TC PDX models. Phase I/II trials with RG7388, known as idasanutlin, and several other MDM2 inhibitors, including AMG-232, ds3032b and ALRN-6924 are ongoing in solid tumour patients. Functional p53 protein is necessary for MDM2 inhibitor efficacy making TC patients eligible for this therapy since *TP53* mutations are rarely observed in these patients^23–25^. Phase I clinical trials with the mTORC1/2 inhibitor TAK-228 (MLN0128) showed good tolerability of the inhibitor alone^51^ and in combination with other drugs^52^. Phase II trials with TAK-228 are currently on going. Anti-tumour effects induced by RG7388 or AZD8055 have been observed in the two cisplatin-sensitive TC PDX models. Surprisingly, neither AZD8055 nor RG7388 treatment did alter tumour growth in TC4. This interesting PDX model, derived from a patient refractory to cisplatin treatment, now appears to be a multi-drug resistant model as demonstrated by the lack of response to cisplatin but also to MDM2 or mTORC1/2 inhibitors. Further investigation of how to circumvent this broad drug resistance of TC4 may feed into the design of new drug combinations for patients with cisplatin refractory/relapsed TC. The mutation observed in *RBBP8/CtIP* might render PDX model TC4 highly sensitive to PARP inhibitors, and could therefore be a potential therapeutic target for this resistant PDX model. Immunotherapy for TC patients is also being investigated as a potential targeted treatment in clinical trials (NCT03081923, NCT03158064). However, due to the lack of a functional immune system, TC PDX models are not suitable to study the pre-clinical efficacy of immunotherapy.

In summary, we have established three PDX models of testicular cancer and charactersied them at the histological, genomic and transcriptional level. These models showed accurate drug sensitivity prediction for cisplatin and can now be used for mechanistic studies and pre-clinical validation of novel therapeutic strategies in testicular cancer.

## Methods

### Establishing of tumour xenografts

Patients of which tumour material was used were all participants of OncoLifeS, a large data biobank at the University Medical Center of Groningen (UMCG), University of Groningen, the Netherlands. The study design was approved by the scientific committee of OncoLifeS. The OncoLifeS databiobank has been approved by the Ethics Committee of the UMCG. All patients provided written informed consent for participation in OncoLifeS. All animal experiments were approved by the Institutional Animal Care and Use Committee of the University of Groningen (Groningen, the Netherlands) in accordance with the approved guideline “code of practice: animal experiments in cancer research” (Netherlands Inspectorate for Health Protection, Commodities and Veterinary Public Health, 1999). Mice were kept under pathogen free conditions and received sterilized food and water ad libitum. Subcutaneous TC PDX models were established as described previously^12^. Taking into account the heterogeneity of TC, sampling of the tumour was assisted by a pathologist who aimed at selecting different areas guided by macroscopic examination. Histology of each tumour piece was evaluated subsequently by an experienced oncological pathologist who determined TC components, based on the WHO classification of tumours of the urinary system and male genital organs^53^. In short, primary tumour or biopsy material was cut into ~ 3 × 3 × 3 mm^3^ pieces and implanted on both flanks of 4–12 week old male NOD.Cg-Prkdcscid Il2rgtm1Wjl/SzJ (NSG) mice (internal breed, Central Animal Facility, University Medical Centre Groningen). When sufficient material was available, the tumour was also biobanked in liquid nitrogen using freeze media: FCS (Life Technologies, Waltham, MA, USA) with 5% DMSO (Sigma, St. Lois, MO, USA), paraffin embedded and snap frozen. Tumour growth was monitored by caliper measurements once a week and tumour volume was calculated using the following formula (width^2^ x length)/2. Once tumour volume reached > 1500–2500 mm^3^, mice were sacrificed and tumours harvested. These tumours were used for immediate re-implantation into the next generation (P0, P1, P2), as well as biobanked in liquid nitrogen using freeze media, paraffin embedded and snap frozen.

Histology of each tumour piece was evaluated subsequently by an experienced oncological pathologist who determined TC components. P0 (passage 0): First generation/passage mouse implanted with the original human specimen. P1 (passage 1): Second generation/passage mouse implanted with the specimen from P0. P2 (passage 2): Third generation/passage mouse implanted with the specimen from P1.

### Immunohistochemistry

Formalin-fixed and paraffin embedded material was cut into 4 µm sections and mounted on glass slides. Hematoxylin and eosin (H&E) staining was used to look at tumour histology. Immunohistochemical (IHC) staining was done for Ki-67, cyclophilin A, AFP and cleaved caspase-3. Tissue slides were deparaffinised in xylene and rehydrated in ethanol. Antigen retrieval was done for 15 min as listed in Supplementary Table 5. Endogenous peroxidase was blocked for 30 min with 0.3% H_2_O_2_. Tissue slides were then incubated with the primary antibodies diluted in PBS with 1% BSA (Serva, Heidelberg, Germany) for 1 h at room temperature or at 4 °C overnight (Suppl. Table 5). Slides were stained with HRP labelled secondary antibodies (DAKO, Santa Clara, CA, USA), staining was visualized by DAB and counterstained with hematoxylin.

Stained sections were scanned using the NanoZoomer 2.0-HT multi slide scanner (Hamamatsu, Hamamatsu City, Japan). Automated scoring of scanned images was done with QuPath, an open source, digital image analysis software^54^. Scoring of Ki-67 and cleaved caspase-3 were done in different ways. For Ki-67 scoring, QuPath software was trained using an automated cell detection classifier to separate tumour from stromal background populations. The simple tissue detection command was applied to detect the total tissue area of each image. Then the cell detection command, followed by the automated cell detection classifier was used, automatically calculating the percentage of Ki-67-positive tumour cells. For cleaved caspase-3, the first step was using the simple tissue detection command. Next, the positive pixel count command was applied, giving the positive pixel count. Data are represented as positive pixel count / area µm^2^.

### DNA and RNA isolation of PDX tumours

DNA and RNA from PDX tumours of different generations were isolated from snap frozen tumour tissue. Frozen section slides were used for quantifying the amount of vital tumour cells. Cryostat sections of 10 um were cut (~ 25/tumour) for DNA and RNA isolation, with additional 4 µm sections for H&E staining to estimate tumour/normal cell percentage. Simultaneous isolation of DNA and RNA from the same sample was done using the AllPrep DNA/RNA mini kit (Qiagen, Hilden, Germany).

### Whole-exome sequencing

Paired-end sequencing (150 bp) was performed on two generations PDX tumours (P0, P2) and the primary tumour of TC1. Samples were prepared for hybridization capture using the Agilent SureSelectXT Clinical Research Exome v2 kit. Briefly, genomic DNA was fragmented and adapters were added, followed by PCR amplification. The quality and yield after sample preparation were measured with the Fragment Analyzer. Libraries were sequenced using Illumina HiSeq 4000 according to manufacturer’s protocols. A concentration of 3.0 nM of DNA was used and sequencing was performed by GenomeScan (Leiden, the Netherlands). Primary data analysis and quality score calculations were performed with RTA v2.7.7 and Bcl2fastq v2.20 (Illumina, San Diego, CA, USA). On average, 45–55 million paired reads were generated per sample. Prior to alignment, the reads were trimmed for adapter sequences using Trimmomatic v0.30^55^. Reads were mapped to both the mouse (GRCm38.p4) and human (GRCh37.75) reference to segregate mouse and human reads using BBMap (v36.62; https://sourceforge.net/projects/bbmap/). Sequencing data were further analysed in collaboration with the Genetics department of the UMCG using an in-house bioinformatics pipeline. Variant calling was performed using GATK v3.8^56^ and Freebayes v1.1.0^57^. A summary of the QC data is given in supplementary Table 6. Variant filtering and somatic mutation identification were performed as previously described^58^. Variants with a minimum read depth of 10 or at least 5 altered reads were considered for further analysis. In addition, variants with CADD scores < 15 were considered benign. Variants were manually assessed in Integrative Genomics Viewer (IGV). The OncoKB Cancer Gene List (https://oncokb.org/cancerGenes) was checked for presence of tumour suppressor- and oncogenes. All tumours were sequenced without matched DNA from normal tissue.

### Single nucleotide polymorphism genotyping

The Infinium Global Screening Array-24 v1.0 BeadChip from Illumina containing ~ 700.000 SNPs to determine copy number variation was used for genome-wide SNP genotyping of the different generations PDX tumours of TC1, TC4 and TC5. DNA processing, tagging and hybridization to the CHIP were performed according to the manufacturer's protocol (Illumina). Primary assessment and SNP call rate quality control of SNP intensity output files were performed using GenomeStudio software (Illumina). Samples passed the inclusion quality control criteria, including call rates > 95%. Further analysis was performed with Nexus Copy number software (BioDiscovery, El Segundo, CA, USA) to generate copy number alteration (CNA) profiles. Quantitative CNA correlative analysis of the different PDX passages were performed as described previously^12^.

### RNA sequencing

The NEBNext Ultra Directional RNA Library Prep Kit (New England Biolabs (NEB), Ipswich, MA, USA), was used to process the samples. Ribosomal RNA was depleted from total RNA using the rRNA depletion kit (NEB). After fragmentation of the RNA, cDNA synthesis was performed. cDNA was ligated with the sequencing adapters followed by a PCR amplification. The quality and yield after sample preparation were measured with the Fragment Analyzer. Clustering and DNA sequencing using the NovaSeq 6000 (Illumina) was performed according to manufacturer's protocols. A concentration of 1.5 nM of DNA was used. Sequencing was performed by GenomeScan (Leiden, the Netherlands).

Primary data analysis and quality score calculations were performed with RTA3 and Bcl2fastq v2.20 (Illumina). Prior to alignment, the reads were trimmed for adapter sequences using Trimmomatic v0.30^55^. Reads were mapped to both the mouse (GRCm38.p4) and human (GRCh37.75) reference to segregate mouse and human reads using BBMap (v36.62; https://sourceforge.net/projects/bbmap/) Alignment of reads was done by mapping to the human reference sequence (GRCh37.75) using Tophat v2.0.14^59^ with default settings and were quantified on gene level. A summary of the QC data is given in supplementary Table 7. For differential expression analysis, read counts were loaded into the DESeq2 (v1.14.1)^60^ package within the R platform (v3.3.0). Average hierarchical clustering of differentially expressed genes and visualization was performed within R (v3.4.1) using gplots package^61^. Clusters were defined visually setting the cut off at 0.7. In order to analyse the differentially expressed genes (DEGs) at the functional level, we used the PANTHER classification system to obtain the enriched biological processes (BPs), molecular function (MF) and cellular compartment (CC)^62^. Correlative analysis on DEGs was performed using complete hierarchical clustering of Spearman correlation metrics. All computations and heatmap generation were performed using R (v3.4.1).

### Efficacy studies of standard of care and novel therapeutics

Mice were implanted with PDX tumours as described above. When tumours demonstrated sustained growth, mice were randomized into vehicle control or treatment groups (n = 3–7 mice/group). Cisplatin (Accord Healthcare, London, UK) (1 mg/kg or 4 mg/kg) or vehicle (saline) was administered weekly via intraperitoneal injection. AZD8055 (Axon MedChem, Groningen, Netherlands) (2.7 mg/kg or 10 mg/kg in 10% DMSO, 40% Polyethylene glycol 300 (Sigma)) or vehicle was administered daily via intraperitoneal injection. RG7388 (Selleckchem, Munich, Germany) (50 mg/kg or 75 mg/kg in 2% hydroxypropylcellulose (Sigma), 0.2% Tween-80 (Sigma)) or vehicle was administered daily via oral gavage. Tumour growth was assessed 3 times a week using caliper measurements. All mice were sacrificed after 21 days of treatment. For future analysis the tumours were resected and paraffin embedded. Tumour volumes are plotted as mean ± SEM for all groups. Two-way ANOVA with Dunnett post hoc test was used to determine the significance of all pair-wise comparisons using GraphPad Prism.

### Evaluation of AFP serum levels in PDX models

During the cisplatin efficacy study, blood samples from PDX mice were collected at the start and end of treatment via retro-orbital bleeding. After blood coagulation, samples were centrifuged for 15 min at 1500 rpm and serum was collected. To measure AFP levels in mice serum, an ELISA kit for detection of AFP in human serum of plasma was used according to manufacturer’s instructions (NovaTec Immundiagnostica, Dietzenbach, Germany). Absorbance was measured at 450 nm using an iMARK microplate absorbance reader (Bio-Rad, Hercules, CA, USA). AFP concentrations were determined against a standard curve provided by the ELISA kit. All samples were measured in duplicates.

## Supplementary information

Supplementary Information 1.

Supplementary Table 3.

Supplementary Table 4.

## Data Availability

The PDX models will be added to the collection of the EuroPDX consortium. The data that support the findings of this study are openly available in Gene Expression Omnibus (GEO) database and Sequence Read Archive (SRA), reference numbers PRJNA639088, GSE152270.
