# Supplementary material for: Establishment and characterisation of testicular cancer patient-derived xenograft models for preclinical evaluation of novel therapeutic strategies
Source: Sci Rep. 2020 Nov 3;10:18938. doi: 10.1038/s41598-020-75518-3 (PMC7641131; doi:10.1038/s41598-020-75518-3)
Supplement: Supplementary file 1 — Supplementary Information 1. [file 41598_2020_75518_MOESM1_ESM.pdf]

Establishment and characterization of testicular cancer patient-derived xenograft models for preclinical evaluation of novel therapeutic strategies

Gerda de Vries[1,#], Ximena Rosas-Plaza[1,#], Gert Jan Meersma[1], Vincent C. Leeuwenburgh[1], Klaas Kok[2], Albert J.H. Suurmeijer[3], Marcel A. van Vugt[1], Jourik A. Gietema[1], and Steven deJong[1,*].

[1]Department of Medical Oncology and [3]Department of Pathology, Cancer Research Center Groningen, University of Groningen, University Medical Center Groningen, Groningen, The Netherlands. [2]Department of Genetics, University of Groningen, University Medical Center Groningen, Groningen, The Netherlands.

[#] These authors contributed equally to this work.

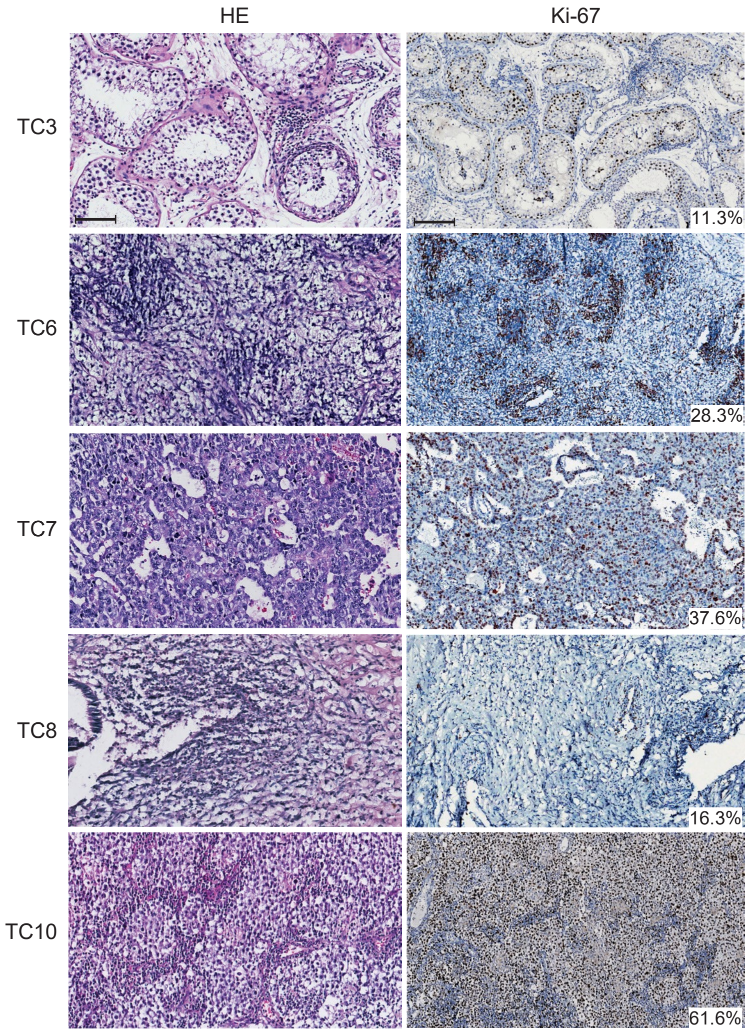

Supplementary Figure 1. *Histopathological characteristics of tumour that failed to engraft.* HE and Ki-67 staining at 20X and 10X magnification of patient tissue that failed to engraft as PDX. Insertions in Ki-67 staining state proliferation index of each tumour. Scale bars for the HE pictures represent 100 μM, and for Ki-67 pictures scale bars represent 200 μM.

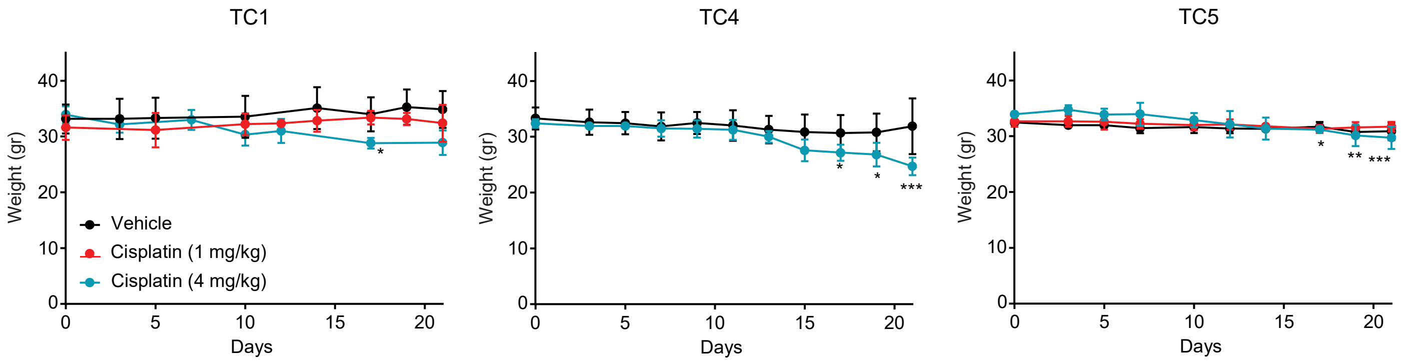

Supplementary Figure 2. *Effect of cisplatin treatment on mouse weight over time.* Mouse weight was measured 3 times a week while animals were in treatment. Animals were treated with cisplatin for 21 days according to the experimental set-up of Figure 6a.

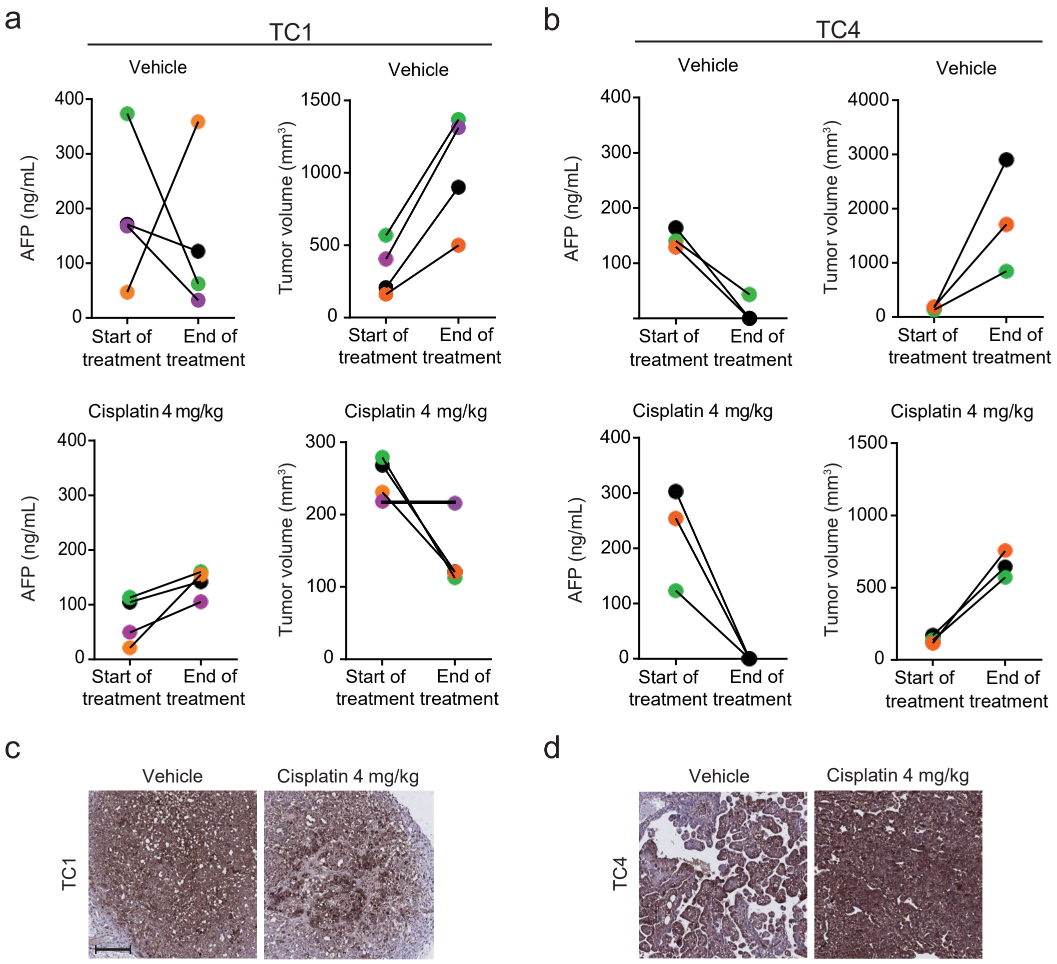

Supplementary Figure 3. *Comparison of biomarker levels and tumour volume.* **a**, AFP levels detected in mouse serum from PDX TC1 at start and end of either vehicle or cisplatin treatment (4 mg/kg), and matching tumour volumes. Coloured dots indicate paired samples from individual mice. **b**, AFP levels and tumour volumes from PDX TC4 at start and end of treatment as in (**a**). Coloured dots indicate paired samples from individual mice. **c** and **d**, Representative images of tumours shown in (**a**) and (**b**) respectively at 10x magnification stained for AFP. Scale bars represent 200 μM.

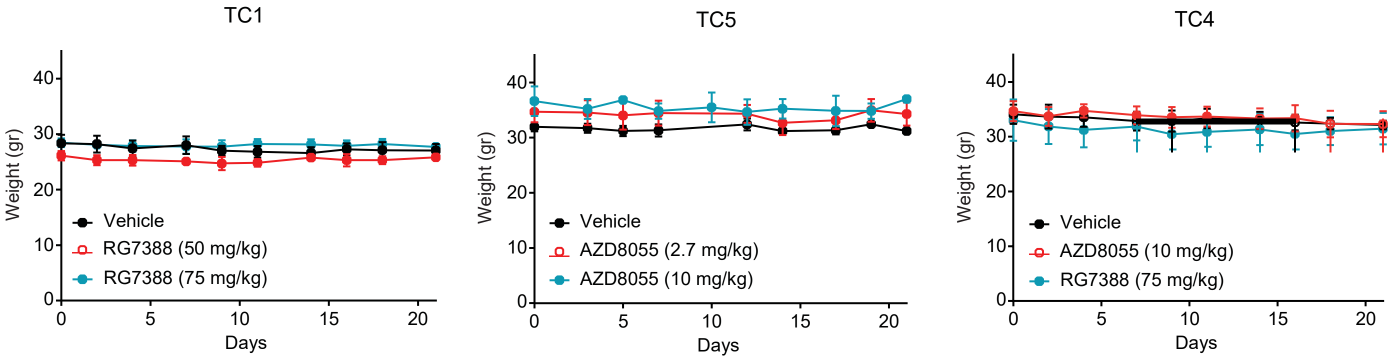

Supplementary Figure 4. *Effect of AZD8055 or RG7388 treatment on mouse weight over time.* Mouse weight was measured 3 times a week while animals were in treatment. Animals were treated with AZD8055 (TC5 and TC4) or RG7388 (TC1 and TC4) for 21 days according to the experimental set-up of Figure 7a, c, e and g.

Supplementary table 1. Latency time and take rate of fresh and biobanked TC tissue

		Fresh implantation		Biobanked tissue	
PDX model	Passage	Median latency time (days)	Take rate (%)	Median latency time (days)	Take rate (%)
TC1	P0	55	3/6 (50)	7	1/4 (25)
	P1	35	3/4 (75)	25	7/10 (70)
	P2	23	4/6 (67)	/	/
TC4	P0	21	1/1 (100)	/	/
	P1	/	/	/	/
	P2	12	6/6 (100)	34	8/14 (57)
TC5	P0	29	5/6 (83)	/	/
	P1	/	/	22	9/10 (90)
	P2	19	4/4 (100)	/	/

Supplementary table 2. Splitting of human and mouse reads

Sample	Organism	% unique reads	% ambiguous reads
TC1.patient	Human (GRCh37.75)	99.72	0.03
	Mouse (GRCm38.p4)	0.01	0.03
TC1.P0	Human	96.83	0.04
	Mouse	2.91	0.04
TC1.P2	Human	91.67	0.06
	Mouse	8.04	0.06
TC4.P0	Human	98.45	0.03
	Mouse	1.29	0.03
TC4.P2	Human	98.55	0.03
	Mouse	1.18	0.03
TC5.P0	Human	95.65	0.05
	Mouse	4.08	0.05
TC5.P2	Human	95.70	0.05
	Mouse	4.00	0.05

The majority of reads (91.7-99.7%) were human.

Supplementary table 5. Antibodies and antigen retrieval used for immunohistochemical stainings

Antigen	Antigen retrieval	Company (catalogue nb.)	Dilution	Incubation	Detection
Ki-67	Tris/EDTA (pH 9.0)	DAKO (M7240)	1:350	60 minutes 20 ºC	RAMhrp - GARhrp
P53	Tris/EDTA (pH 9.0)	DAKO (M7001)	1:1000	60 minutes 20 ºC	RAMhrp - GARhrp
AFP*		Ventana (760-2603)			
Cyclophillin A	EDTA (pH 8.0)	Cell signaling (51418)	1:500	60 minutes 20 ºC	GARhrp - RAGhrp
Cleaved caspase-3	EDTA (pH 8.0)	Cell signaling (9661)	1:100	60 minutes 20 ºC	GARhrp - RAGhrp

Abbreviations: RAMhrp = Rabbit -anti-Mouse horseradish peroxidase, GARhrp = Goat-anti-Rabbit horseradish peroxidase, o/n = overnight. * Run on Ventana Benchmark Ultra automated system

Supplementary Table 6. Whole exome sequencing quality report

Sample Name	Total reads x 10^6	Reads aligned	Unique reads aligned	Mean target coverage	Target Bases 10x
TC1.Pt	56.6	100%	80.9%	65.0	96%
TC1.P0	46.6	100%	83.9%	58.4	95%
TC1.P2	49.0	100%	82.4%	59.7	94%
TC4.P0	59.1	100%	84.1%	72.1	96%
TC4.P2	48.3	100%	85.3%	59.8	93%
TC5.P0	58.2	100%	84.4%	70.0	96%

Supplementary Table 7. RNA sequencing quality report

Sample Name	Total reads x 10^6	Filtered reads x 10^6	Total mapping	Uniquely mapped reads
TC1.Pt	33.1	32.3	83.1%	78.1%
TC1.P0	18.5	16.8	85.1%	78.9%
TC1.P2	31.9	26.9	84.4%	77.2%
TC4.P2	15.4	14.6	88.1%	81.3%
TC5.P0	15.5	14.2	87.7%	83.0%
TC5.P2	47.7	39.3	82.7%	76.4%
